# Anatomical Variations of the Aortic Arch: A Computerized Tomography-Based Study

**DOI:** 10.7759/cureus.13115

**Published:** 2021-02-03

**Authors:** Uma Pandalai, Minnie Pillay, Srikanth Moorthy, Tintu T Sukumaran, Swapna Ramakrishnan, Asha Gopalakrishnan, Anandhu Krishnan Gopalakrishna Pillai

**Affiliations:** 1 Anatomy, University of Hafr Al Batin, Riyadh, SAU; 2 Anatomy, Amrita Institute of Medical Sciences and Research, Kochi, IND; 3 Radiology, Amrita Institute of Medical Sciences and Research, Kochi, IND

**Keywords:** aortic arch, aortic branches, aortic variations, aortic anomalies, ct aortography

## Abstract

Introduction

Despite adequate preparation and meticulous pre-operative assessment, variations of the vascular anatomy of the aortic arch may lead to clinical dilemmas. In the present era, with the easy availability of imaging facilities, various anatomical variations can be found out prior to an interventional procedure. However, there are many countries including India where such facilities may still be not widely available. The purpose of this study was to assess the prevalence of these anatomical variants in patients undergoing Computerised Tomography (CT) chest with contrast.

Methods

This observational study involved patients who underwent CT chest with contrast as part of various clinical indications during a three-year period in a tertiary care centre in South India. Variations of the aortic arch and its branching pattern were studied in 4,000 chest CT images of patients referred to the radiology department.

Results

A total of 4,000 patients underwent CT chest with contrast during the study period. Twenty-seven variations were observed in these patients. They included aberrant right subclavian artery in seven patients, bovine arch in one patient, bovine origin of left vertebral artery from arch in one patient, bronchial artery of anomalous origin from arch in one patient, double aortic arch in one patient, and right-sided aortic arch in 16 patients.

Conclusion

The variant anatomy of the aortic arch has tremendous clinical significance, especially from the surgical standpoint. Anatomical variants can also cause difficulty during catheterization while performing endovascular interventions. Given the prevalence demonstrated in our study, imaging may be indicated prior to any procedure involving vascular access in order to prevent unwanted complications.

## Introduction

The anatomic and morphologic variation of the aortic arch (AA) and its branches assumes importance in diagnostic and surgical procedures of the neck and thorax [1]. Radiological evaluation of anatomy and pathology involving the aorta has undergone considerable refinement in recent years [2]. Non-invasive cross-sectional imaging, including ultrasonography, computerised tomography (CT), and magnetic resonance imaging (MRI) have replaced traditional aortography [2]. This has limited the role of catheter angiography, which is now restricted to patients who require catheter-based interventions.

Though there are many studies on the variations of aortic arch and its branching pattern in cadavers, only a few studies are available that are based on CT and from the Indian subcontinent. In this study, we aimed to demonstrate the prevalence of the variations of the arch of aorta and its branching pattern from a relatively large population who underwent CT chest with contrast. 

## Materials and methods

This was a retrospective observational study involving all patients who underwent CT chest with contrast during a three-year period between 2014 to 2017 in a tertiary care centre in South India. Any patient who underwent CT chest with contrast for any indication was included in the study. The data was accessed through the hospital health information system (HIS). Ethical clearance was obtained from the institutional review board. 

Definitions and methods

In our centre, during the study period, all CT scans were performed using a multi-detector (MDCT) device (256-iCT Philips, Amsterdam, The Netherlands). Plain CT chest images, including the root of the neck and upper abdomen, were taken first. It was followed by the injection of iodinated contrast media with an iodine concentration of 350 g/L, which was injected using an 18G intravenous (IV) catheter in adults and appropriately sized smaller gauge IV cannulas in children and infants. Post-contrast arterial phase images were acquired with a delay of 25 seconds covering from the root of the neck to the pancreas. Images were acquired during a single breath-hold. Reconstruction was done at intervals of 5 mm and 1 mm slice thickness in the axial and coronal planes. Images were transferred to a workstation and multiplanar reformatted images were obtained.

Statistical methods

Anatomical variants were identified and expressed in terms of the prevalence of each variant. Chi-square test for trend analysis was performed for gender with a 95% confidence interval. A p-value of less than 0.05 was considered significant. IBM SPSS version 20 (IBM Corp., Armonk, NY, USA) was used for statistical analysis. 

## Results

4,000 patients underwent CT chest with contrast during the study period. A total of 27 variations were observed in the 4,000 CT images, making the prevalence of variations 0.675%.

There were 2,400 (60%) male (M) and 1,600 (40%) female (F) patients. Out of the total 27 variations observed, 16 (59.2%) cases involved males and 11 (40.8%) were females. Chi-square test for trend analysis for correlation of variations with gender was not significant (p=0.503). 

The variations included seven cases (25.9%) of aberrant right subclavian artery (four M, three F), one case of bovine arch (M), one case of bovine origin of left vertebral artery from arch (M), one case of bronchial artery of anomalous origin from arch (M), one case of double aortic arch (F), and 16 cases of right-sided aortic arch (eight M, eight F). 

The variations are summarised in Table 1. 

**Table 1:** Variations of aortic arch and its branching pattern as observed on CT images.

Serial number	Variations observed	Number of cases (n)	Males	Females
1	Aberrant right subclavian artery	7	4	3
2	Bronchial artery of anomalous origin from arch	1	1	-
3	Bovine arch	1	-	1
4	Bovine origin of left vertebral artery from arch	1	1	-
5	Double aortic arch	1	-	1
6	Right-sided aortic arch	16	8	8
	Total	27	14	13

## Discussion

This was a retrospective observational study conducted over three years at a tertiary care centre in South India. Variations of aortic arch occur when the development of normal arterial pattern is disturbed. Aortic arches develop initially by vasculogenesis, soon after the neural crest cells have invaded the early pharyngeal arches. Angiogenic mesenchyme forms the endothelial lining of the vessels, and the neural crest contributes to the outer layer of the walls. The first aortic arch artery is a part of the original vascular circuit that links the truncus arteriosus to the paired dorsal aorta. As the heart descends, the aortic sac gives rise to paired aortic arches at successive caudal levels, which pass lateral to the pharynx to join the dorsal aorta. The persistence of arch arteries that normally disappear, either in part or in full, or disappearance of some arch arteries that should in the normal course persist, leads to the anomalous pattern [3]. 

Variations in the branching pattern of the aortic arch

1. Aberrant Right Subclavian Artery (ARSA)

In the present study, a total of seven cases of ARSA were observed, out of which in five cases the course of ARSA was retro-oesophageal and in two cases retro-tracheal. In this study, four patients were asymptomatic while three had oesophageal and tracheal compression. ARSA is formed by the distal portion of the right dorsal aorta and right seventh intersegmental artery. With the shortening of the aorta between left common carotid and left subclavian arteries, the origin of ARSA settles inferior to that of the left subclavian artery [4]. As its stem derives from the right dorsal aorta, ARSA has to cross the midline in order to reach the right arm. Thus, it takes a retro-tracheal or retro-oesophageal course, or very rarely, passes anterior to the trachea [4].

**Figure 1:**
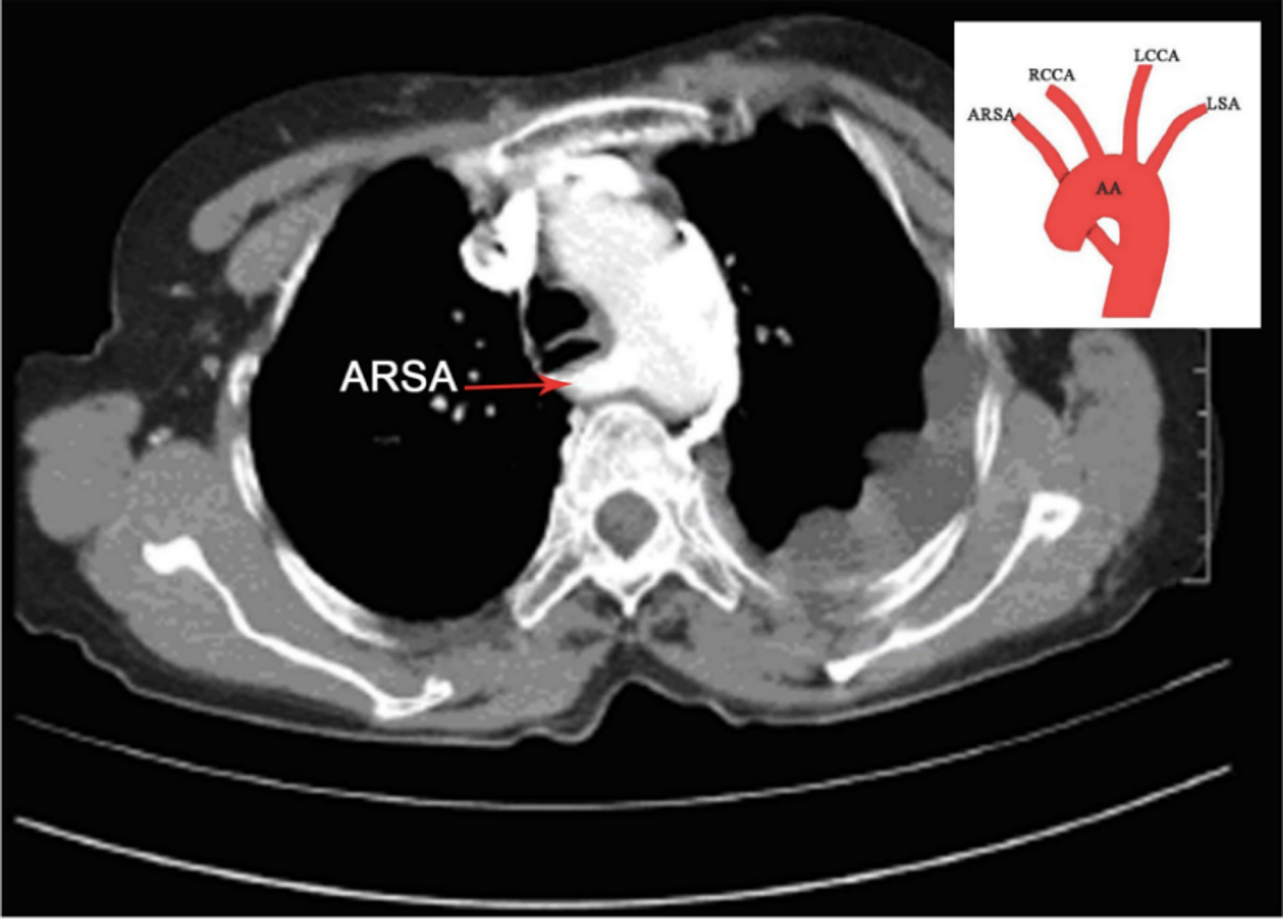
Axial CT chest contrast image showing aberrant right subclavian artery with schematic representation of finding. ARSA: Aberrant Right Subclavian Artery, AA: Aortic Arch, RCCA: Right Common Carotid Artery, LCCA: Left Common Carotid Artery, LSA: Left Subclavian Artery

ARSA could be a potential risk factor for tracheotomy bleeding and its presence may cause unforeseen problems in transradial coronary procedures [5]. According to available literature, only 60% of procedures using a transradial approach were successful in the presence of an arteria lusoria [5]. Borenstein et al. reported that ARSA is associated with an increased incidence of intra-cardiac malformations, and examination of the position of ARSA is likely to become a routine ultrasound marker for chromosomal abnormalities in the second trimester of pregnancy [6]. While the prevalence of ARSA reported in the literature has been highly variable, we observed a prevalence of 0.175%. An illustrative example of ARSA from our study is shown in Figure 1. 

2. Bronchial Artery of Anomalous Origin (BAAO)

The origin of bronchial arteries outside the lower margin of T4 and the upper margin of T6 is considered as the bronchial artery of anomalous origin (BAAO) [7]. BAAO arises from supra-aortic trunks or its branches, aortic arch or its branches, coronary arteries or abdominal aorta. This should be kept in mind while performing diagnostic and therapeutic studies of bronchial circulation in patients, especially in recurring cases of hemoptysis in which the cause of bleeding cannot be ascertained.

In bronchial arterial embolization (BAE) and hemoptysis, BAAO is a causative factor for bleeding. Because of the small diameter, the bronchial arteries are of high resistance and low capacitance, or of low distensible circulation [8]. They undergo smooth muscle wall hypertrophy or dilate to direct more oxygenated blood in ischemic lung tissue in patients with systemic hypoxia, pulmonary infection and various other inflammatory diseases that affect thorax [9]. Abnormal tortuous bronchial arteries manifest in multidetector CT or MRI imaging as nodular and linear enhancing structures that are best viewed on multiplanar or three-dimensional (3D) volumetric images [8]. During imaging interpretation, BAAO may be mistaken for lymph nodes, veins, endobronchial lesions, broncholithesis, or oesophageal enhancement [9]. Cauldwell et al. classified bronchial arteries into nine types based on the number on each side. According to them, in 40% of the cases, there were two bronchial arteries on the left and one on the right. A single bronchial artery was observed bilaterally in 21.3% of the cases, and two bronchial arteries bilaterally in 20.6% cases. In 9.7% of the cases, there was only a single bronchial artery on the left and there were two on the right [10].

**Figure 2:**
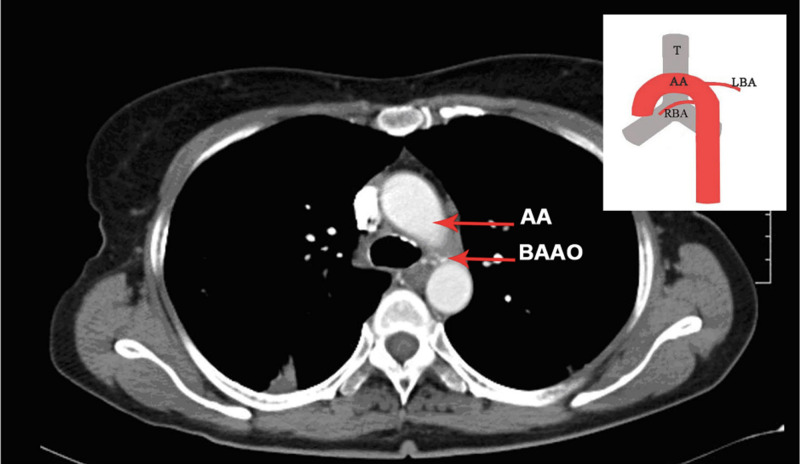
Axial CT chest contrast image showing left bronchial artery of anomalous origin from under surface of arch of aorta at the level of T3 inferior border with schematic representation of finding. BAAO: Bronchial Artery of Anomalous Origin, AA: Aortic Arch, T: Trachea, RBA: Right Bronchial Artery, LBA: Left Bronchial Artery

In the present study, two cases of BAAO (one M, one F] were observed. The first case was BAAO type two of Cauldwell’s classification, where the left bronchial artery was seen arising from the aortic arch (T3 inferior border) and the right from the aorta (T4 superior border). Both arteries were dilated and tortuous. The second case was an incidental finding where a superior bronchial artery/ectopic bronchial artery was noted arising from the internal thoracic artery (T2 inferior border). The artery was dilated and tortuous. Left bronchial artery took origin from descending thoracic aorta. The reported range of prevalence of ectopic bronchial arteries varies from 8.3% to 56% [7,11,12]. An illustrative example of BAAO from our study is shown in Figure 2. 

3. Bovine Arch (BA)

The term 'Bovine arch' refers to a group of congenital variants of aortic branches in which there is an aberrant origin of the left common carotid artery. Its prevalence ranges from 8% to 25% [13]. 

Lippert et al. classified the bovine arch into two types [14]. The left common carotid artery takes origin from the innominate artery with a prevalence of 9% [15]. The other being the commonest with common origin of innominate and left common carotid arteries with the prevalence ranging from 8% to 25% in different ethnic groups [13,15]. In the present study, a similar case was noted as an incidental CT finding.

The precise embryological events underlying the development of common origin of the innominate and left common carotid arteries remain incompletely understood. Normally the proximal part of the left third aortic arch gets absorbed into the left horn of aortic sac [16]. Instead, if it gets absorbed into the right horn of aortic sac, it shows branching pattern variations like the left common carotid artery arising from brachiocephalic trunk [16].

**Figure 3:**
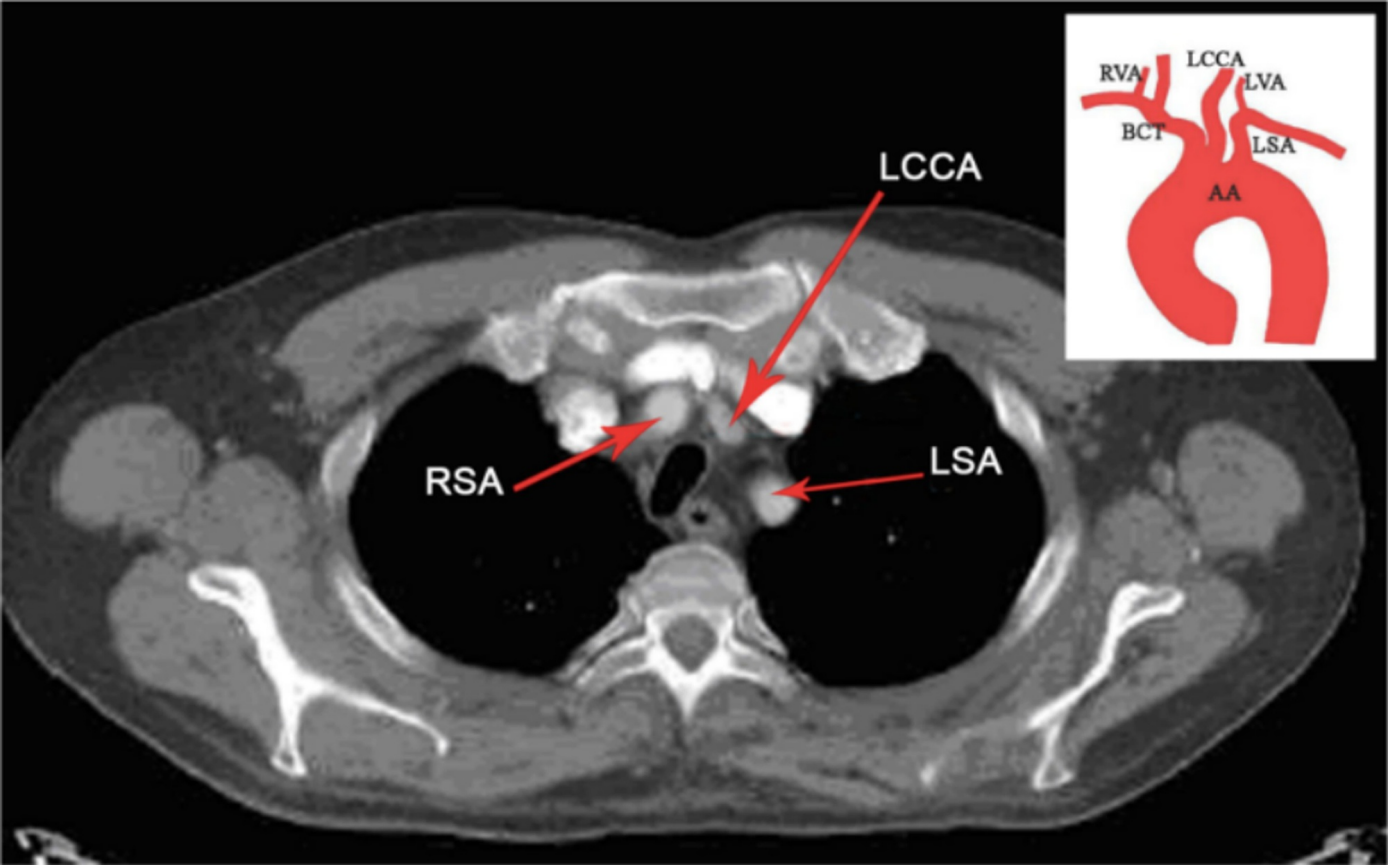
Axial CT chest contrast image showing type 1 bovine arch with schematic representation of finding. AA: Aortic Arch, BCT: Brachiocephalic Trunk, RVA: Right Vertebral Artery, LCCA: Left Common Carotid Artery, LVA: Left Vertebral Artery, LSA: Left Subclavian Artery

BA could be associated with technical failure and neurological complications in carotid stenting procedures. In blunt chest trauma, BA is found to be associated with an increased likelihood of innominate transection at its take-off from the aorta [17]. An illustrative example of BA from our study is shown in Figure 3. 

4. Bovine Origin of Left Vertebral Artery (LVA) from the Aortic Arch

Vertebral arteries (VA) normally arise as branches from the subclavian artery and are important for the posterior cerebral circulation. Prevalence of anomalous origin of the vertebral artery from aortic arch ranges from 1% to 5% [18]. In the present study, the patient had a wide range of cardiovascular malformations like atretic main pulmonary artery, proximal right pulmonary artery stenosis, patent ductus arteriosus, large ventricular and atrial septal defects, single ventricle double superior vena cava, interrupted inferior vena cava, polysplenia, etc.

Lippert et al. classified LVA according to the origin from the aortic arch into eight types: A-H [14]. In the present study, type A of Lippert's classification of LVA was observed in one case. Normally, the first part of the vertebral artery develops from the proximal part of the dorsal branch of the seventh cervical intersegmental artery proximal to post-costal anastomosis. Longitudinal communications of the postcostal anastomoses give rise to the second part. In the present case, the left sixth dorsal intersegmental artery that normally disappears might have persisted as the first part of vertebral artery; hence the left vertebral artery took origin from aortic arch between the left common carotid and left subclavian arteries [14].

**Figure 4:**
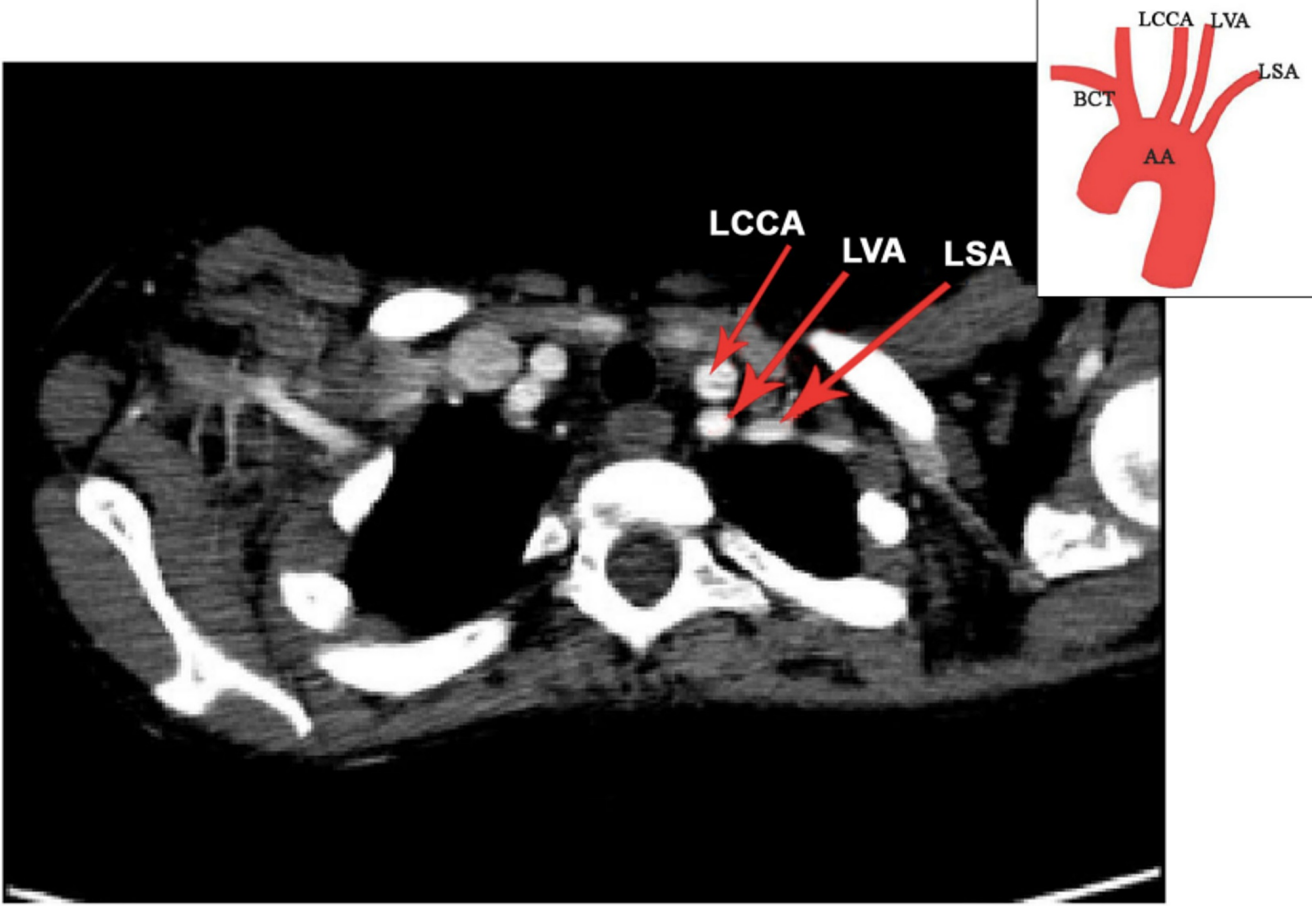
Axial CT chest contrast image showing bovine origin of left vertebral artery with schematic representation of finding. AA: Aortic Arch, BCT: Brachiocephalic Trunk, LCCA: Left Common Carotid Artery, LVA: Left Vertebral Artery, LSA: Left Subclavian Artery

The vertebral arteries provide blood supply to the posterior neck musculature, the rhombencephalon, the mesencephalon, the occipital lobe and part of the temporal lobe of the brain [19]. Variations in the origin of the vertebral artery may lead to altered hemodynamics, predisposing to intracranial aneurysm formation. Therefore, a thorough search for coexisting aneurysms should be undertaken in patients with these anomalies. Endovascular therapy of intracranial aneurysms can be performed before they present clinically as subarachnoid haemorrhages, and thereby decrease morbidity and mortality. An illustrative example of bovine origin of LVA from our study is shown in Figure 4. 

Variations of the actual aortic arch

1. Double Aortic Arch (DAA)

Double aortic arch is a rare congenital anomaly in which two aortic arches form a complete vascular ring that can compress the trachea or oesophagus [20]. DAA accounts for 46-76% of complete vascular rings [21,22]. Double aortic arch appears when the involution of the right fourth arch fails to occur. DAA usually occurs as an isolated lesion, it may be associated with congenital heart diseases such as tetralogy of Fallot (TOF), ventricular septal defect, and transposition of great arteries [23]. DAA can be divided into two types depending upon the patency of two arches. In type one, both arches are patent, and in type two, one arch (usually the left) is atretic [24].

**Figure 5:**
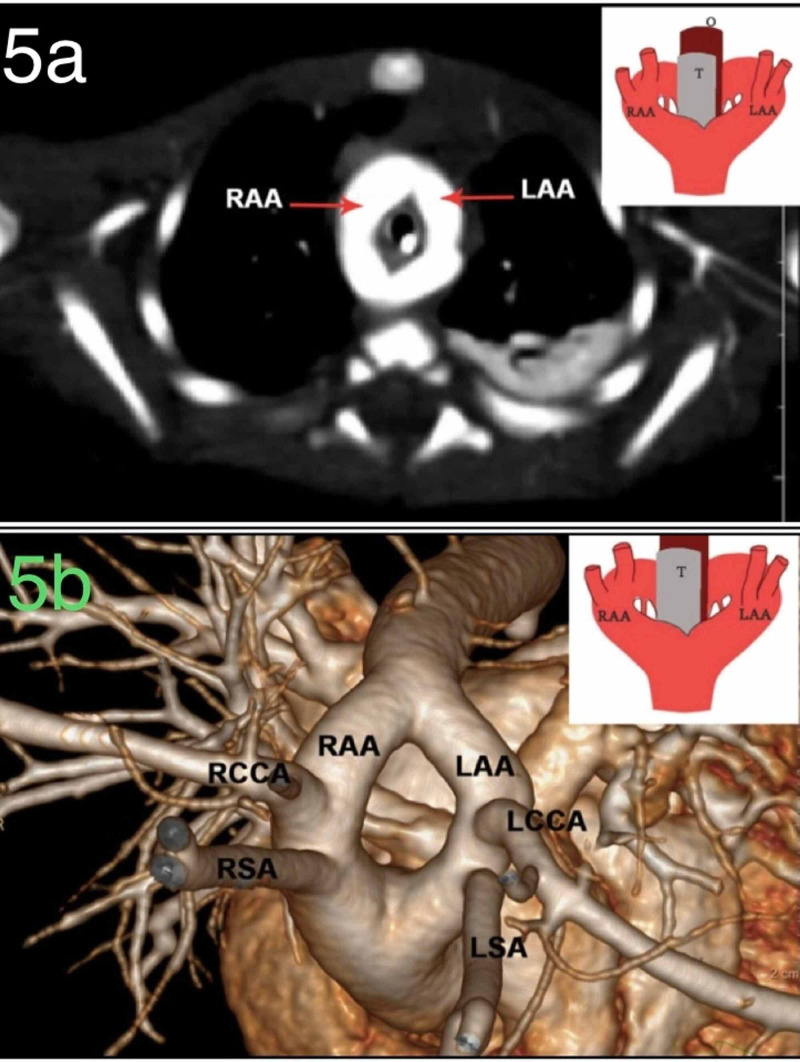
5a: Axial CT Chest contrast image showing double aortic arch with schematic representation of finding. 5b: 3D reconstructed image of double aortic arch with schematic representation of finding. RAA: Right Aortic Arch, LAA: Left Aortic Arch, RCCA: Right Common Carotid Artery, RSA: Right Subclavian Artery, LCCA: Left Common Carotid Artery, LSA: Left Subclavian Artery, T: Trachea; O: Oesophagus

In the present study, a single case of type one DAA was observed. The right arch was found to be the dominant one. Right subclavian and right common carotid were the branches seen arising from the right arch, and left subclavian and left common carotid were branches from the left arch. Other congenital anomalies like situs solitus, subaortic ventricular septal defect, TOF, infundibular pulmonary stenosis, and tracheal stenosis were also observed besides DAA. An illustrative example of DAA from our study is shown in Figure 5. 

2. Right-Sided Aortic Arch (RAA)

Right-sided aortic arch (RAA) which is observed in 0.1% of the adult population is a type of AA variant characterised by the AA coursing to the right of the trachea [25]. In the present study, a total of 16 cases of right-sided aortic arch were observed.

A total of six pairs of aortic arches develop at different stages of organogenesis. If the left fourth arch, which normally persists, disappears and the right, which usually disappears, persists, a right aortic arch results. Approximately 50% of cases of right-sided aortic arches are associated with aberrant left subclavian artery and is generally asymptomatic except when a vascular ring is present [26]. Congenital heart anomalies such as TOF, pulmonary stenosis, ventricular septal defect, and tricuspid atresia occur in almost 80% of cases with right-sided aortic arch. In the present study, a large number of congenital defects were associated with right-sided aortic arch. 

RAA is usually an incidental radiological finding and is asymptomatic. RAA with Kommerell’s diverticulum can present with respiratory symptoms when tracheal compression occurs [27]. In the present study, Kommerell’s diverticulum was observed in two cases. Anomalies of the arch system can give rise to Kommerell's diverticulum, most frequent in right-sided aortic arches with an aberrant left subclavian artery [28]. In Kommerell's diverticulum, the left subclavian artery originates from a diverticulum at the junction of the right descending aorta and the right aortic arch, and it passes obliquely superiorly, posterior to the oesophagus, and towards the left arm. Generally, the diverticulum is well developed, as at the origin of the aberrant left subclavian artery, the fetal ductus arteriosus carries a large volume of blood [29].

In contrast, patients who have TOF and a right-sided aortic arch do not develop an aortic diverticulum [30]. They have low ductal flow during fetal life, as infundibular stenosis tends to limit the normal right-to-left flow. The presence of a ligamentum arteriosum or a left ductus arteriosus between the left pulmonary artery and the left subclavian artery results in a vascular ring. Symptoms of oesophageal or tracheal compressions are often mild or even absent as the ring is generally loose [29]. In the present study, only one case of Kommerell’s diverticulum had tracheal compression, ligamentum arteriosum, and TOF. 

**Figure 6:**
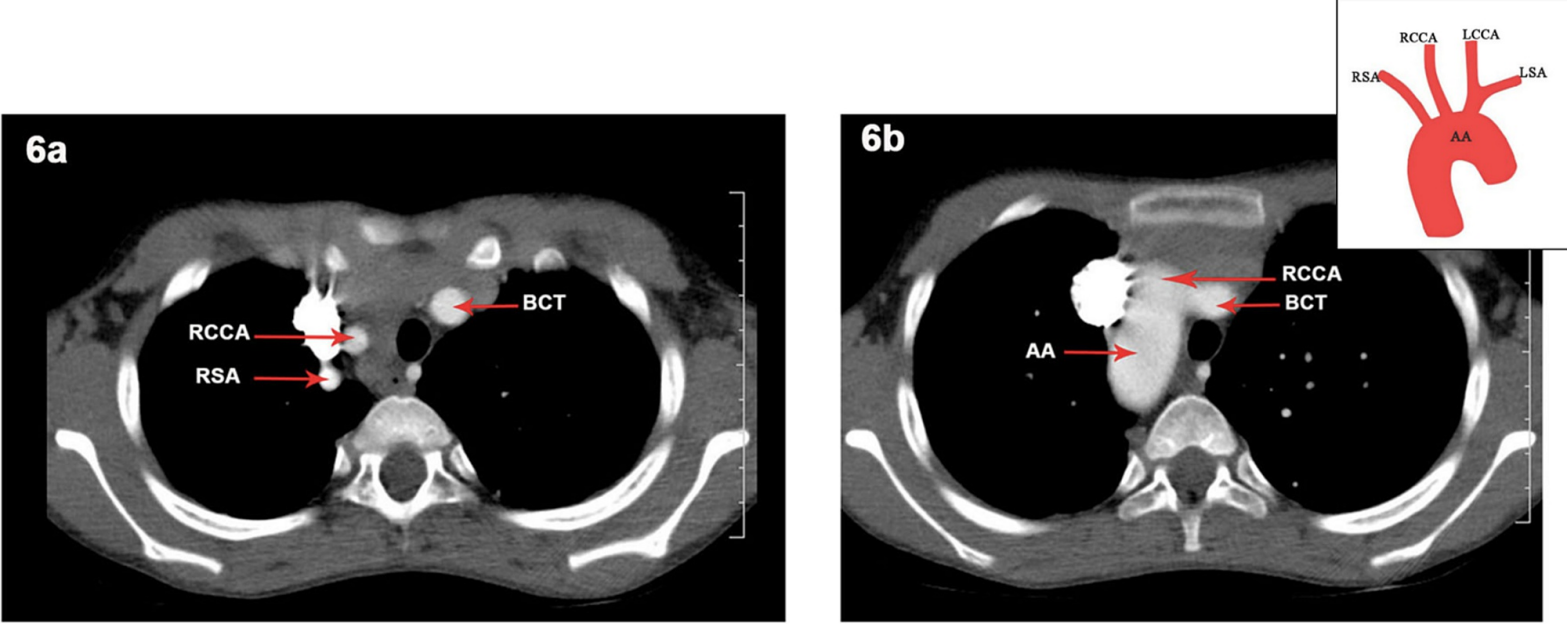
6a: Axial CT chest contrast image showing type I right aortic arch. 6b: Axial CT chest contrast image showing mirror image branching pattern with schematic representation of finding. AA: Aortic Arch, RSA: Right Subclavian Artery, RCCA: Right Common Carotid Artery, BCT: Brachiocephalic Trunk, LCCA: Left Common Carotid Artery, LSA: Left Subclavian Artery

**Figure 7:**
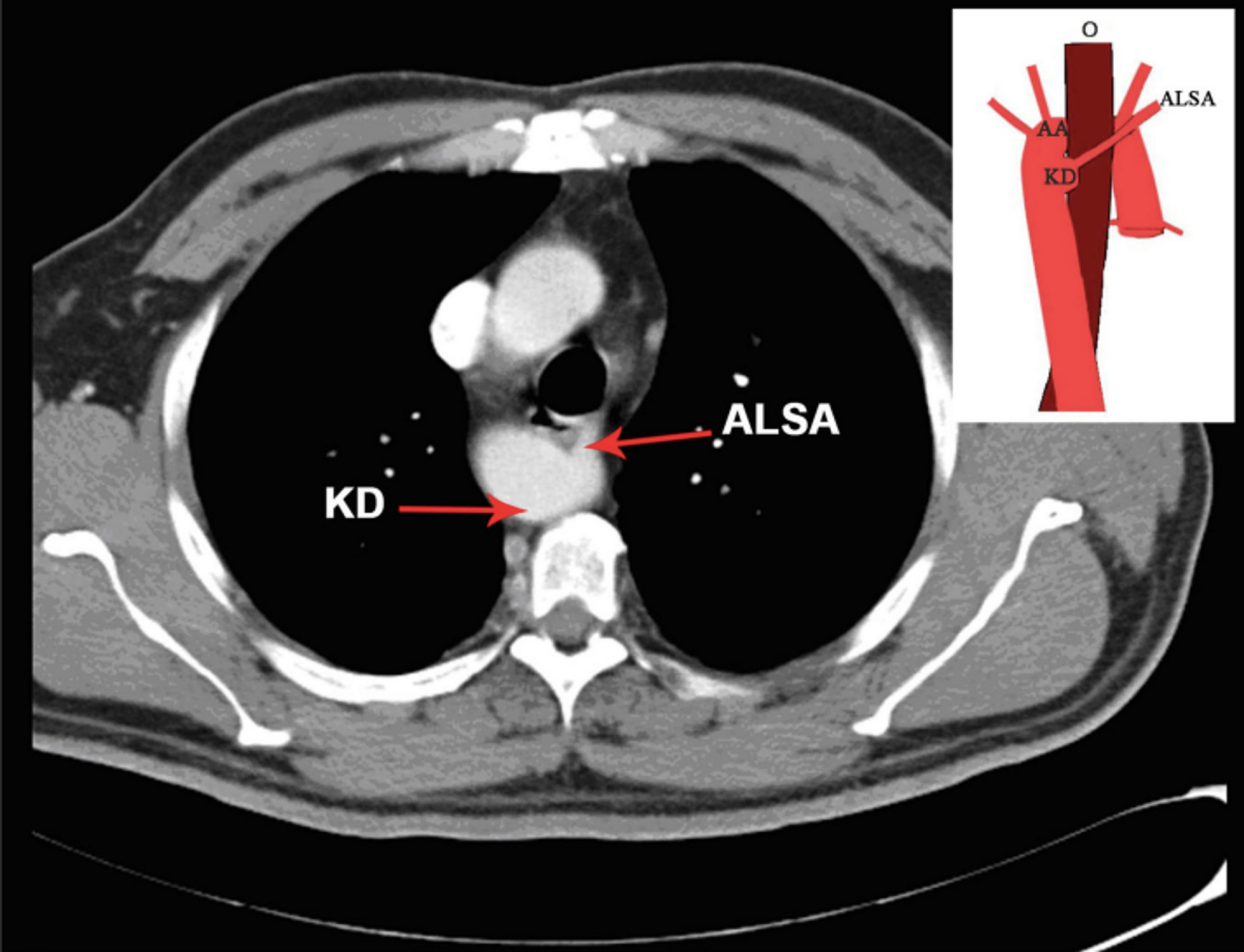
Axial CT chest contrast image depicting right-sided aortic arch with Kommerell with schematic representation of finding. AA: Aortic Arch, KD: Kommerell diverticulum, ALSA: Aberrant Left Subclavian Artery, O: Oesophagus

RAA was the commonest variation in the present study. But since the slice thickness of the axial CT chest contrast images used for the study was mostly 5 mm, variations like BA were difficult to observe. BA can be visualised only in slices with 1 mm thickness, and hence is a limitation of this study. This study has other limitations as well. As it was a retrospective study, a complete clinical profile of the patients with variations in the AA could not be captured. The prevalence reported may not be the true prevalence as only patients who underwent imaging of the chest were included in the study, which could have led to an amount of selection bias. Finally, follow up of the patients or the past medical history of the patients in the study was not possible due to its cross-sectional nature and similar longitudinal studies may be warranted to address this deficiency. Illustrative examples of RAA from our study are shown in Figures 6, 7. 

## Conclusions

The variant anatomy of the aortic arch has tremendous clinical significance, especially from the surgical stand point. Anatomical variants can also cause difficulty during catheterization while performing endo-vascular interventions.

Given the prevalence demonstrated in our study, imaging should be included prior to any procedure involving vascular access in order to prevent complications. More research is warranted in quantifying the burden of aortic arch variation in the population, especially so from the Indian subcontinent. 

## References

[1] Nayak SR, Pai MM, Prabhu LV, D'Costa S, Shetty P (2006). Anatomical organization of aortic arch variations in India: embryological basis and review. J Vasc Bras.

[2] Sparks AR, Johnson PL, Meyer MC (2002). Imaging of abdominal aortic aneurysms. Am Fam Physician.

[3] (2008). Gray’s Anatomy. The Anatomical Basis of Clinical Practice. https://scholar.google.com/scholar?hl=en&as_sdt=0%2C5&q=Standring+S%2C+Borley+NR%2C+Collins+P%2C+Crossman+AR%2C+Gatzoulis+MA%2C+Healy+JC%2C+et+al.%2C+editors.+Gray’s+Anatomy.+The+Anatomical+Basis+of+Clinical+Practice.+40th%C2%A0edn.+New+York%3A+Churchill+Livingstone%3B2008.&btnG=.

[4] Sukumaran TT, Pillay M, Gopalakrishnan A (2015). An anomalous right subclavian artery with a retrotracheal course: a case report. J Clin Diagn Res.

[5] Valsecchi O, Vassileva A, Musumeci G (2006). Failure of transradial approach during coronary interventions: anatomic considerations. Catheter Cardiovasc Interv.2006.

[6] Borenstein M, Minekawa R, Zidere V, Nicolaides KH, Allan LD (2010). Aberrant right subclavian artery at 16 to 23+ 6 weeks of gestation: a marker for chromosomal abnormality. Ultrasound Obstet Gynecol.

[7] Amrhein TJ, Kim C, Smith TP, Washington L (2011). Bronchial artery arising from the left vertebral artery: case report and review of the literature. J Clin Imaging Sci.

[8] Osiro S, Wear C, Hudson R, Ma XX, Zurada A, Michalak M, Loukas M (2012). A friend to the airways: a review of the emerging clinical importance of the bronchial arterial circulation. Surg Radiol Anat.

[9] Walker CM, Rosado-de-Christenson ML, Martínez-Jiménez S, Kunin JR, Wible BC (2015). Bronchial arteries: anatomy, function, hypertrophy, and anomalies. Radiographics.

[10] Cauldwell EW, Siekert RG, Lininger RE (1948). The bronchial arteries: an anatomic study of 150 human cadavers. Surg Gynecol Obstet.

[11] Battal B, Akgun V, Karaman B, Bolzar U, Tasar M (2011). Normal anatomical features and variations of bronchial arteries: an analysis with 64-detector-row computed tomographic angiography. J Comput Assist Tomogr.

[12] Hartmann IJ, Remy-Jardin M, Menchini L, Teisseire A, Khalil C, Remy J (2007). Ectopic origin of bronchial arteries: assessment with multidetector helical CT angiography. Radiol.

[13] Baadh AS, Rockman CB, Mitnick RJ, Lim RP (2014). Bovine arch and carotid artery atherosclerosis: are they related?. Clin Imaging.

[14] Lippert H, Pabst R (1985). Arterial variations in man: classification and frequency.

[15] De Garis CF, Black IH, Riemenschneider EA (1933). Patterns of the aortic arch in American White and Negro stocks, with comparative notes on certain other mammals. J Anat.

[16] Moore KL, Persaud TVN (2003). The Developing Human: Clinically Oriented Embryology. Philadelphia: Elsevier Science.

[17] Graham JM, Feliciano DV, Mattox KL, Beall AC (1982). Innominate vascular injury. J Trauma.

[18] Vorster W, Du Plooy PT, Meiring JH (1998). Abnormal origin of internal thoracic and vertebral arteries. Clin Anat.

[19] Satti SR, Cernigilia A, Koenigsberg RA (2007). Cervical vertebral artery variations-An anatomical study. Am J Neuroradiol.

[20] Nagre SW, Kulkarni DV (2015). Double aortic arch surgery. Indian J Vasc Endovasc Surg.

[21] Riemenschneider TA, Allen HD, Gutgesell HP (1995). Moss and Adams' Heart Disease in Infants, Children, and Adolescents: Including the Fetus and Young Adult. https://scholar.google.com/scholar?hl=en&as_sdt=0%2C5&q=Emmanouilides+GC%2C+Allen+HD%2C+Gutgesell+HP%2C+et+al.+Heart+Disease+in+Infants%2CChildren+and+Adolescents+Including+the+Fetus+and+Young+Adult.+Baltimore%3A+Williams+%26+Wilkins%3B+1995&btnG=.

[22] Van Son JA, Julsrud PR, Hagler DJ (1993). Surgical treatment of vascular rings: the Mayo Clinic experience. Mayo Clin Proc.

[23] Yıldırım SV, Yıldırım A (2017). Truncus arteriosus with double aortic arch: a rare association. Turk J Pediatr.

[24] Griswold HE, Young MD (1949). Double aortic arch: report of two cases and review of literature. Pediatrics.

[25] Hori D, Tanaka M, Yamaguchi A, Adachi H (2009). Type A aortic dissection, right-sided aortic arch, and thoracic aortic aneurysm. Asian Cardiovasc Thorac Ann.

[26] Yang MH, Weng ZC, Weng YG, Chang HH (2009). A right-sided aortic arch with Kommerell's diverticulum of the aberrant left subclavian artery presenting with syncope. J Chin Med Asso.

[27] Backer CL, Monge MC, Popescu AR, Eltayeb OM, Rastatter JC, Rigsby CK (2016). Vascular rings. Semin Pediatr Surg.

[28] Tyczyński P, Michałowska I, Wolny R (2017). Left aberrant subclavian artery. Systematic study in adult patients. Int J Cardiol.

[29] van Son JA, Konstantinov IE (2002). Burckhard F Kommerell and Kommerell's diverticulum. Tex Heart Inst J.

[30] Velasquez G, Nath PH, Castaneda-Zuniga WR, Amplatz K, Formanek A (1980). Aberrant left subclavian artery in tetralogy of Fallot. Am J Cardiol.

